# A novel method for quantifying human disturbances: A case study of Huaihe River Basin, China

**DOI:** 10.3389/fpubh.2022.1120576

**Published:** 2023-01-09

**Authors:** Haoran Wang, Mengdi Zhang, Chuanying Wang, Kaiyue Wang, Yunkai Zhou, Wei Sun

**Affiliations:** ^1^College of Geography and Environmental Science, Henan University, Kaifeng, China; ^2^Key Laboratory of Geospatial Technology for the Middle and Lower Yellow River Regions, Henan University, Ministry of Education, Kaifeng, China; ^3^Henan Key Laboratory of Earth System Observation and Modeling, Henan University, Kaifeng, China; ^4^Key Laboratory of Watershed Geographic Sciences, Nanjing Institute of Geography and Limnology, Chinese Academy of Sciences, Nanjing, China

**Keywords:** human disturbances, HDI, land use naturalness, resource consumption, pollutant emissions, spatiotemporal change, Huaihe River Basin

## Abstract

Human disturbances have become the main factors affecting the ecological environment. Therefore, evaluating the intensity of human disturbances is of great significance for ensuring effective regional conservation and ecosystem management. In this study, we constructed a novel method to quantify human disturbances based on three components of human disturbances into three types, namely naturalness transformation, natural resource consumption, and pollutant emissions. These components were quantified using the land use naturalness index (LNI), resource consumption index (RCI), and pollution emission index (PEI). Based on these three indicators, the human disturbances index (HDI) was calculated to reflect the intensity of human disturbances. In addition, remote sensing (RS), geographic information system (GIS), and multisource data were combined in the HDI method, taking into account the temporal variability of input parameters to achieve more convenient and comprehensive dynamic monitoring and evaluation of human disturbances. The applicability and effectiveness of the HDI method were assessed in the Huaihe River Basin, China. The obtained results revealed an increase and decrease in the intensities of human disturbances in the Huaihe River Basin from 1990 to 2005 and from 2010 to 2018, respectively. In addition, areas with a high level of human disturbances in the 1990–2005 period were mainly concentrated in the agricultural and industrial areas, while those in the 2010–2018 period were mainly observed in urban areas. This change was mainly due to a decrease in the pollutant emission amounts from agricultural and industrial lands and a marked increase in resource consumption in urban areas. This study provides theoretical guidance for regional conservation in the Huaihe River Basin and a new method for quantifying human disturbances.

## 1. Introduction

Human activities have exerted considerable anthropogenic influences on the earth system since the Anthropocene (1–3). Indeed, the increase in human activities through the rapid growth of population and urbanization has gradually become an important driving factor affecting the ecological environment on a global scale (4–7). According to previous studies, over 75 and 90% of the earth's land surface and riverine systems, respectively, have been altered as a result of human activities (8–12), resulting in a series of eco-environmental problems, such as global warming, water pollution, and land degradation (13–15). The influence of human activities on the environment resulted in a high ecological risk, thereby threatening human survival (16, 17). Therefore, quantifying human disturbances is of great significance to better understand the impacts of human activities on the ecosystem, which is essential for ensuring ecological protection, environmental management, and sustainable development of human society.

Human disturbances have been mainly reflected by various environmental indicators from different perspectives in the initial research stage. Some researchers have assessed the intensities of anthropogenic disturbance by investigating biological indicators and pollution discharges to the water bodies (18–22). However, others have used the species diversity to reflect the intensities of human disturbance in the forest ecosystem (23, 24). Hemeroby is a monitoring indicator, proposed for the first time by Jalas (25) to detect the intensity of human disturbance in forest ecosystems, then has been extensively applied by several researchers to evaluate the human disturbance in various types of ecosystems (10, 19, 26). In addition, numerous evaluation methods for assessing human disturbances have been proposed in recent years, shifting gradually the investigation of human disturbances from qualitative description to quantitative analysis. Indeed, with the development of Geographic Information System (GIS) and Remote Sensing (RS) technologies, data acquisition has become more diversified, rapid, and convenient, offering multi-source data and developing GIS-and RS-based quantitative evaluation methods for assessing human disturbances that integrate GIS and RS technologies (27–30). Many scholars have used land use data obtained from remote sensing images, as well as socio-economic and ground survey data, to analyze the intensity of one or multiple types of human disturbances in certain regions (26, 31–34). On the other hand, Brown and Vivas (3) developed Landscape Development Intensity (LDI) index using energy theory to measure the impact of human disturbances in wetlands. Whereas Sanderson et al. (2) used four types of variable data, namely population density, land transformation, accessibility, and electrical power infrastructure, to map the human footprint as an indicator of human disturbances. However, to date, quantitative research on human disturbances has been mostly based on biased single or multiple indicators using individual or partial components, which are not enough for comprehensively and accurately evaluating the intensities of human disturbances in the ecosystems. Besides the limited applicability of the developed evaluation methods in other areas due to spatial differences in environmental factors, the possibility that the same type of human disturbance source may exhibit different temporal intensities is not considered in the evaluation methods. Therefore, more effective evaluation methods of human disturbances need to be developed based on more comprehensive indicator systems that reflect human activities, taking into account the variability of indicators.

The Huaihe River Basin is an important region for the socioeconomic development of China due to its favorable environment and abundant natural resources, producing 1/6 of the country's agricultural production and serving as an important coal and electricity base in Eastern China (35, 36). In 2018, the Chinese government released the “Huaihe River Economic Belt Development Plan,” which emphasized the excellent location and important status of the Huaihe River Basin, planning to accelerate the development of the Huaihe Ecological and Economic Belt to enhance ecological protection (15). However, the population density in the Huaihe River Basin is substantially higher than the national average population density, resulting in considerable pressure on the natural resources and serious water, air, and soil pollution from industrial pollutants. In general, human disturbances in the Huaihe River Basin are strong and frequent, resulting in severe constraints on its sustainable development.

In this study, multi-source data were used to develop a new method of human disturbances to achieve more convenient and comprehensive dynamic monitoring and evaluation of human disturbances. The human disturbances were classified into three types: land use naturalness transformation, resource consumption, and pollutant emissions. In this study, RS, GIS, and multi-source data were combined to establish the land use naturalness index (LNI), resource consumption index (RCI), and pollution emission index (PEI) to measure three types of human disturbances, taking into account the temporal variability of indicator parameters. Based on these three indicators, human disturbances index (HDI) was calculated to determine the intensities of human disturbances. The applicability and effectiveness of HDI, as well as the spatiotemporal changes in human disturbances, were assessed in the Huaihe River Basin.

## 2. Conceptual framework

Lands are used by humans to obtain produce material products and services through economic activities (3, 30). Different land use types are affected by human activities in different ways and to varying intensities, resulting in different ecological processes and environmental quality. Similar to Ehrlich‘s classic IPAT formula (human impact, population, affluence, and technology), in which human impacts on Earth are equal population size times affluence (interpreted as energy available per person) times technology (37). The number of people in a given area is commonly considered the main factor affecting natural ecosystems, with higher population densities resulting in greater degrees of influence on natural ecosystems (38). Changes in energy source structure are responsible for many significant changes in human disturbances (2). Indeed, fossil fuels and electrical power affect substantially the environment (39). At the same time, air and water pollutant emissions from human production on the supra-regional scale and daily human activities can alter considerably the natural characteristics of the environment (3, 40, 41). In general, the ecosystem is under land conversion pressure, resource consumption, and pollutant emissions caused by human activities.

**Figure 1:**
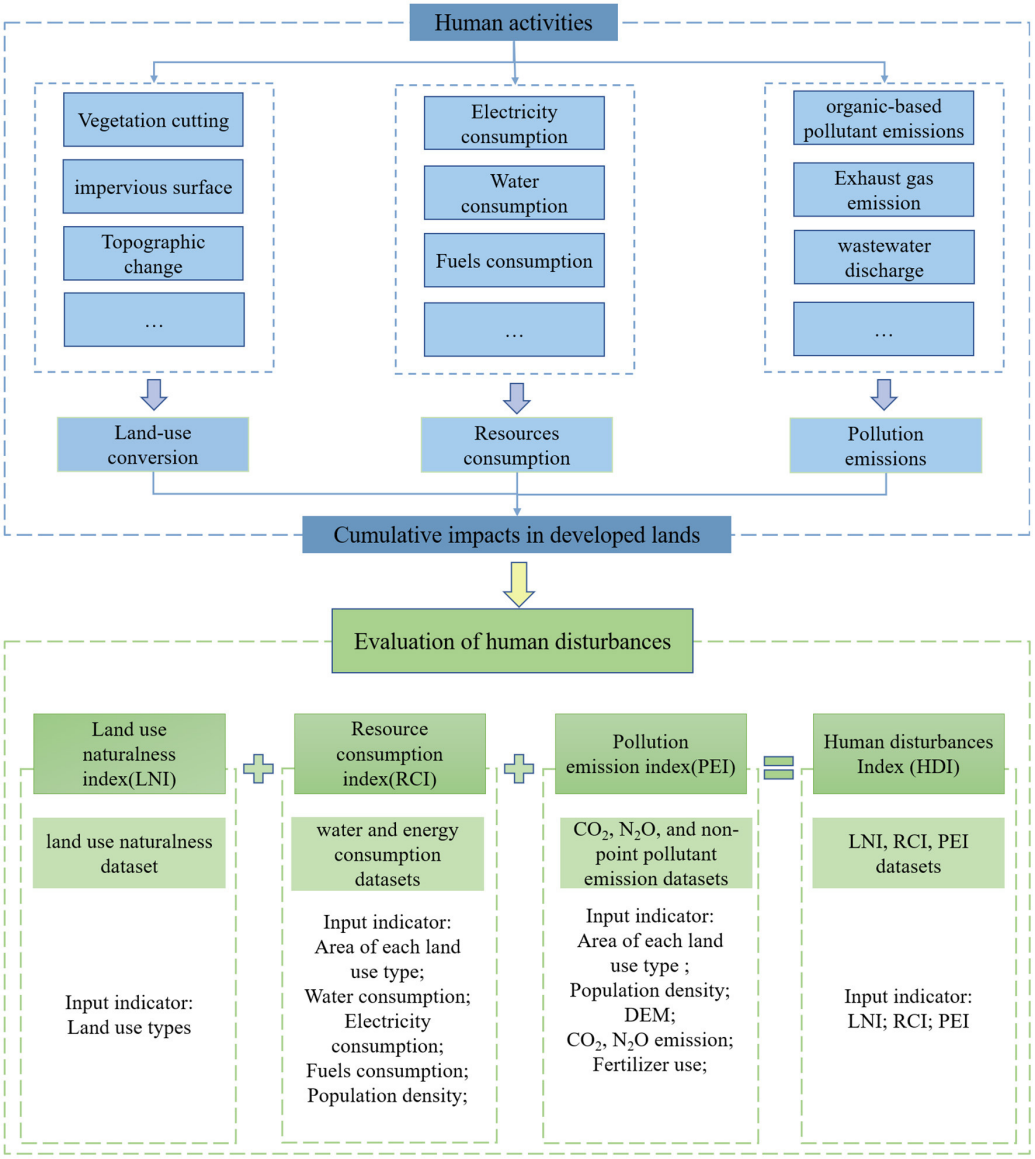
Conceptual framework of HDI.

In this study, the factors of human disturbances on land units were classified into three types, namely land use naturalness transformation, resource consumption, and pollutants emission, then quantified using LNI, RCI, and PEI, respectively. Based on these three indicators, HDI was calculated to reflect the intensities of human disturbances on land units. Six variable data were selected in this study to calculate the intensities of three types of human disturbances according to their coverage, availability, and relevance. Land use naturalness dataset was used to calculate LNI; water and energy consumption datasets were used to calculate RCI; CO_2_, N_2_O, and non-point pollutant emission datasets were used to calculate PEI. The detailed HDI framework flowchart is shown in Figure 1.

## 3. Calculations of human disturbance indices

### 3.1. Land use naturalness index (LNI)

Naturalness is defined as the preservation of natural attributes of the ecosystem (28). Several studies have employed land use transformation to measure the impacts of human disturbances on land naturalness (42, 43) without appropriate classifications. Based on the concept of land use naturalness, Human Activity Intensity of Land Surface (HAILS) was proposed to measure the impacts of human activities on land surface (44), providing a clear scoring system. Therefore, the HAILS evaluation method was applied in this study to assign values to the naturalness of various land uses (Table 1) and calculate the LNI values according to the land use types of the study area. In addition, the subsequent RCI and PEI calculations were also based on this land use classification.

**Table 1:** Classification of land use naturalness.

**Levels**	**Categories**	**Classification signs**	**Scores**
		Natural cover of the land surface does not change and is not used	0
Second level	CS1	Natural cover of land surface does not change but is used	0.67
	CS2	Natural cover of land surface changes and perennial crops are planted	1.33
	CS3	Natural cover of land surface changes and annual crops are planted	2
First level	FCS1	Natural cover of land surface changes	2
	FCS2	There are artificial insulation layers on the land surface. Water could be exchanged. The exchanges of nutrients, air, and heat are impeded	2
	FCS3	There are artificial insulation layers on the land surface. Nutrients could be exchanged. The exchanges of water, air, and heat are impeded	2
	FCS4	There are artificial insulation layers on the land surface. Air could be exchanged. The exchanges of water, nutrients, and heat are impeded	2
	FCS5	There are artificial insulation layers on the land surface. Heat could be exchanged. The exchanges of water, air, and nutrients are impeded	2
		There are artificial insulation layers on the land surface. The exchanges of water, nutrients, air, and heat are impeded	2

### 3.2. Resource consumption index

Previous studies have used DMSP-OLS night light data or calculated the consumption of non-renewable energy resources based on Energy Theory to measure the impacts of human disturbances on the environment (45, 46). However, these methods are limited by data availability and have poor comparability over long-term time scales. LDI considers water and non-renewable resource consumption, allowing for a comprehensive consideration of resource consumption (3). However, the calculations of energy consumption in different land use types and the attributions of hierarchical values of the LDI method are based on statistical methods to calculate energy consumption on different land use types without taking into account the temporal variation in the attributed hierarchical values, thereby restricting the applicability of the method in other regions worldwide.

In this study, the residents' consumption of non-renewable resources and the non-renewable resources consumption per unit area of each land use type were considered according to the calculation method of LDI, then water consumption and energy consumption were divided into two datasets for separate analysis. The non-renewable resource consumption per unit area of different land uses in the study area was calculated for each period based on energy and water consumption statistical data in different periods. In addition, electricity and fossil fuel consumption were converted into standard coal consumption to measure the non-renewable resource consumption. The consumption of residents was calculated using population density statistics and per capita consumption for urban and rural populations, respectively. The water and energy consumption datasets were obtained for different periods by overlay processing in ArcGIS.

### 3.3. Pollutant emission index

Several researchers have devoted considerable attention to point source pollution, resulting in effective control and management of this pollution type, while non-point source pollution still has negative impacts on the natural environment (47–49). Indeed, considerable amounts of airborne and non-point source pollutants have been released into the atmosphere and surface water bodies, respectively, making it challenging to measure their impacts on the natural environment due to the complexity of their dynamics (21, 50). Most studies have simulated non-point source pollution using semi-empirical and physically-based models, such as SWAT, EcoHAT, and HSPF. However, these models require large amounts of data for their calibration and validation (51–53), restricting their applicability in some regions.

In this study, the constantly updated global CO_2_ and N_2_O emission data, published by the Emissions Database for Global Atmospheric Research (EDGAR), were used to measure the impacts of airborne pollutants, including all fossil CO_2_ and N_2_O sources (e.g., fossil fuel combustion, non-metallic mineral processes, agricultural liming, and solvent uses). The CO_2_ and N_2_O emission data were separated into two datasets for separate scoring and analysis.

The assessment of non-point pollution in water bodies is a complex and time-consuming task. The potential non-point pollution index (PNPI) is a GIS tool designed to assess the global pressure of different land uses on rivers and other surface water bodies using simple input data (47). The pollution emission per unit area of each land use type was calculated using statistical data, while the pollution emissions from residential areas were determined using population density and per capita emission data. Finally, the total pollution emission was calculated to determine the land cover indicator (LCI) of PNPI, then PNPI was determined according to Munafo et al. (47). The final obtained result is the non-point source pollution datasets.

### 3.4. Human disturbances index

In this study, the six datasets were overlayed in one map projection using ArcGIS, then standardized scores from 0 (low contribution) to 10 (high contribution) were attributed to reflect the estimated contribution to human disturbances. The natural breaks classification in ArcGIS was used to better classify similar values and maximize the variance between classes. Therefore, based on information from each data during the study period, the natural breaks classification was used to standardize the attributed values to resource consumption and pollutant emissions. The LNI, RCI, and PEI results were obtained by summing the human disturbance scores for each of the six datasets with the same weight. Afterward, stack the three disturbance layers with the same weight were stacked to compute the HDI. High and low HDI values indicate high and low levels of human disturbances, respectively. To further reflect the differences in human disturbances, the HDI values were classified into 10 levels using equal interval classification in ArcGIS (Table 2).


(1) $$\begin{array}{l} LNI+RCI+PEI=HDI \end{array}$$

where *HDI* denotes the human disturbance index of the study area; *LNI* denotes the land use naturalness index of the study area; *RCI* denotes the resource consumption index of the study area; *PEI* denotes the pollution emission index of the study area.

**Table 2:** Classification of HDI values.

**Human disturbance levels**	**I**	**II**	**III**	**IV**	**V**	**VI**	**VII**	**VIII**	**IX**	**X**
HDI	0–6	7–12	13–18	19–24	25–30	31–36	37–42	43–48	49–54	55–60

## 4. Case study

### 4.1. Description of the study area

**Figure 2:**
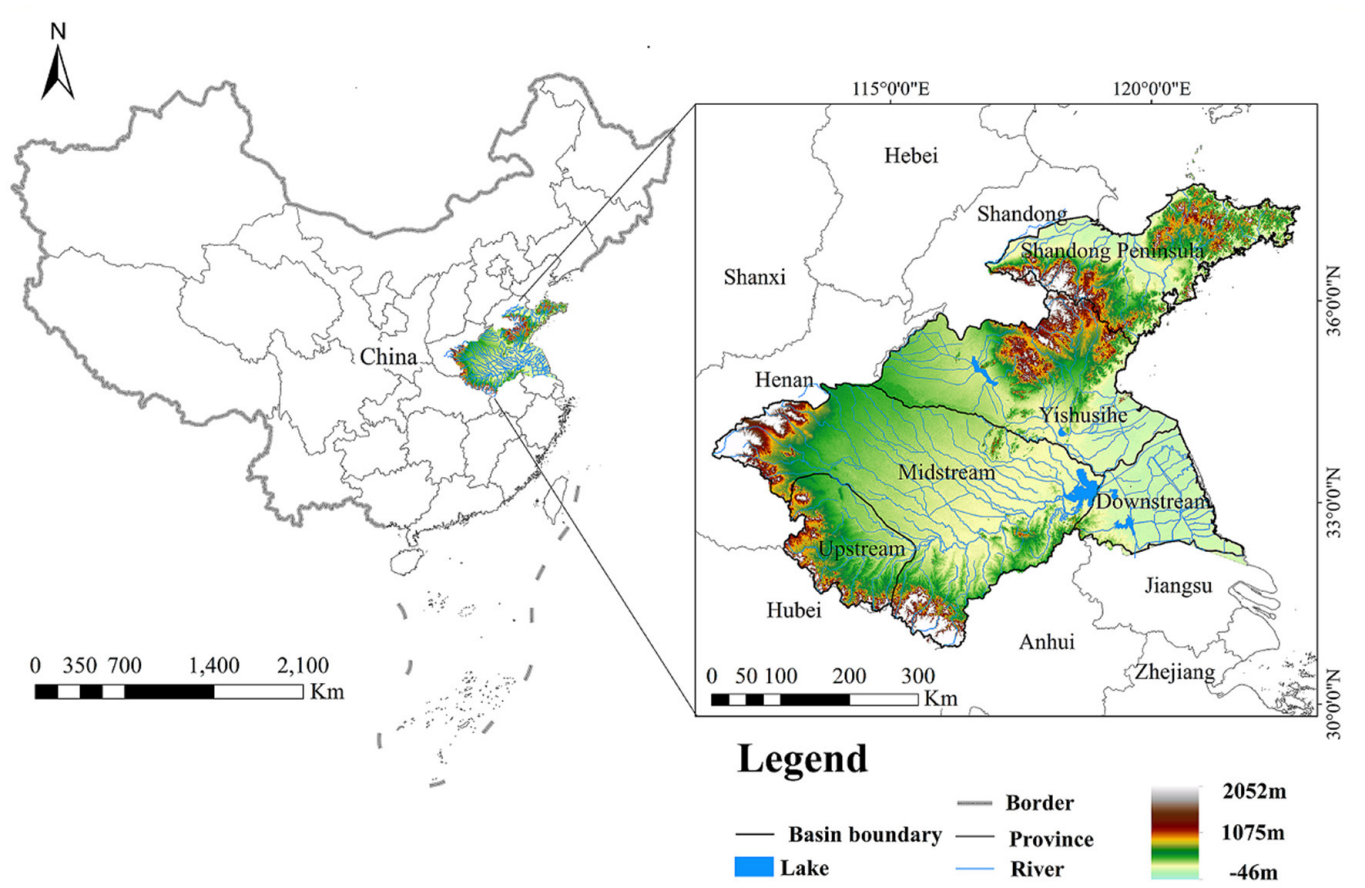
Geographic location of the Huaihe River Basin.

The Huaihe River Basin is located in Eastern China between 30°55′-37°50′ N and 111°55′-122°42′E, covering a total area of ~3.3 × 10^5^ km^2^, including Henan, Hubei, Anhui, Jiangsu, and Shandong Provinces (Figure 2). The study area is separated into five secondary basins, namely upstream, midstream, downstream, Yishusihe, and Shandong Peninsula (15, 54). The mainstream of the Huaihe River is derived from Tongbaishan Mountain in Henan Province, China, flowing eastward into the Yellow Sea (55). On the other hand, the topography of the study area is dominated by low hills in the western and north-eastern parts of the basin and plains, accounting for 1/3 and 2/3 of the total area, respectively (56). The mean annual precipitation and mean annual temperature of Huaihe River Basin are 883 mm and 13.7°C, respectively (57), providing suitable environmental conditions. In addition, the Huaihe River Basin is an important industrial and agricultural production base in China, with a population density of 600 people/km^2^, which is substantially higher than the national population density (148 people/km^2^) (36). Ecosystems in the study area have been under great pressure due to intensive resource exploitation (56).

### 4.2. Data sources and preprocessing

**Figure 3:**
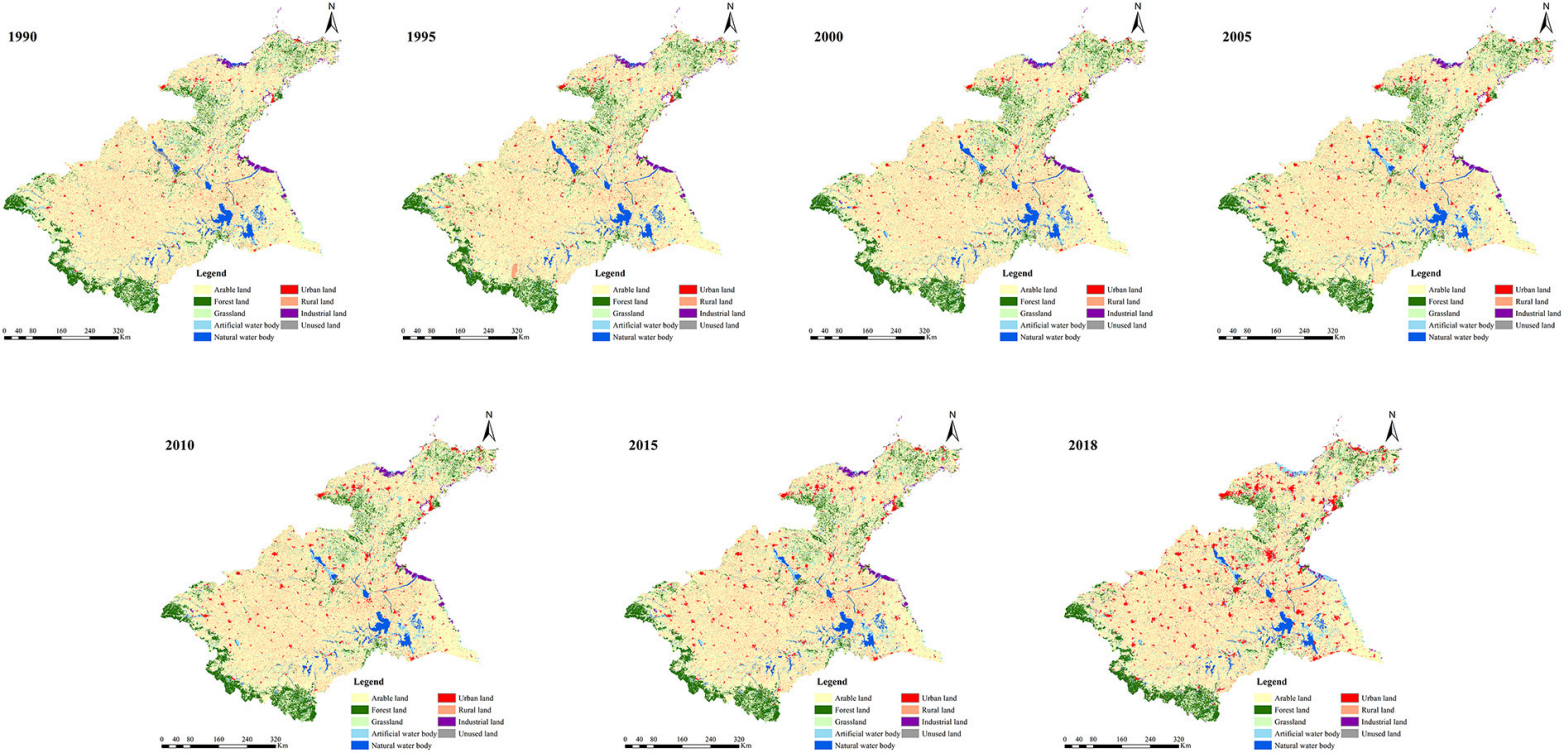
Temporal changes in land use types of the Huaihe River Basin over the 1990–2018 period.

Land use data (spatial resolution of 1 km) for 1990, 1995, 2000, 2005, 2010, 2015, and 2018, and population density data (spatial resolution of 1 km) were obtained from the Resource and Environmental Science Data Center of the Chinese Academy of Sciences. The land use data were generated by processing Landsat remote sensing images, showing overall accuracies >90% (http://www.resdc.cn, accessed on 11 December 2021). We used ArcGIS 10.3 software to classify land use data into nine types, namely arable land, forest land, grassland, artificial water body, natural water body, urban land, rural land, industrial land, and unused land (Figure 3). In addition, soil texture data were obtained from the National Earth System Science Data Center, National Science & Technology Infrastructure of China (http://www.geodata.cn, accessed on 13 December 2021). While digital elevation model (DEM) data (spatial resolution of 1 km, accessed on 11 December 2021) were downloaded from the geospatial data cloud platform (http://www.gscloud.cn, accessed on 11 April 2022). Water consumption data were obtained from the water resources bulletin of the Huaihe River Basin and Shandong Peninsula (http://www.hrc.gov.cn, accessed on 11 April 2022). Electricity and fossil fuel consumption data were derived from the national and provincial statistical yearbook (https://data.cnki.net/Yearbook, accessed on 10 April 2022) of China. CO_2_ and N_2_O emission data were obtained from EDGAR (https://edgar.jrc.ec.europa.eu/, accessed on 11 April 2022).

#### 4.2.1. Land use naturalness index of the Huaihe River Basin

The naturalness of the Huaihe River Basin was obtained in this study (Table 3) based on to the land use type and naturalness classification results (Figure 3 and Table 1).

**Table 3:** Land use naturalness index of the different land use types in the Huaihe River Basin.

**Land use types**	**Classification signs**	**LNI**
Arable land	Natural cover of land surface changes and annual crops are planted	2
Forest land	Natural cover of land surface does not change but is used	0.67
Grassland	Natural cover of land surface does not change but is used	1.33
Artificial water body	There are artificial insulation layers on the surface. Water could be exchanged. The exchanges of nutrients, air, and heat are impeded	6
Natural water body	Natural cover of the land surface does not change and is not used	0
Urban land	There are artificial insulation layers on the surface. Water could be exchanged. The exchanges of nutrients, air, water, and heat are impeded	10
Rural land	There are artificial insulation layers on the surface. Water could be exchanged. The exchanges of nutrients, air, water, and heat are impeded	10
Industrial land	There are artificial insulation layers on the surface. Water could be exchanged. The exchanges of nutrients, air, water, and heat are impeded	10
Unused land	Natural cover of the land surface does not change and is not used	0

#### 4.2.2. Resource consumption index of the Huaihe River Basin

According to the Huaihe River Basin and Shandong Peninsula water resources bulletin, water consumption was classified into six main indicators, namely agriculture, industry, urban residents, rural residents, forestry and husbandry, and urban public. Water consumption per unit area of each land use type was computed independently using land use data, while that for forest land and grassland was calculated using forestry and husbandry data. The water consumption by urban and rural residents was calculated using population density statistics and per capita consumption for urban and rural populations, respectively. The water consumption datasets were obtained for different periods using overlay processing in ArcGIS.

On the other hand, the energy consumption per unit of each land use type and per capita of urban and rural residents were calculated in this study based on the land use data and total energy consumption in different provinces. In addition, because energy consumption for transportation is not limited to roadways, the energy consumption per unit of transportation in urban, rural, and industrial lands was calculated. The temporal energy consumption datasets were obtained for different periods using overlay processing in ArcGIS.

Because of the small area of the Huaihe River Basin in Hubei Province, only Henan, Anhui, Jiangsu, and Shandong Provinces were considered in the calculation. All the resource consumption statistical data used were preprocessed to calculate the 5-year average consumption value, except those from 2016 to 2018, which were used to calculate the 3-year average consumption value.

#### 4.2.3. Pollution emission index of the Huaihe River Basin

In this study, the CO_2_ and N_2_O emission data in the Huaihe River Basin were extracted using the extract by mask tool in ArcGIS software then the kriging interpolation was used to improve the spatial resolution to 1 km. The 5-year average CO_2_ and N_2_O emission values were calculated and considered in this study.

The National Water Environment Capacity Verification Manual, issued by the Chinese government, pointed out that non-point source pollution in China is primarily found in rural and arable areas, where high chemical oxygen demand (COD) and nitrogen oxides levels were observed. Therefore, the values of non-point source pollution were set to 0 for all land use types except for arable and rural lands. The nitrogen oxides emission was used instead of non-point source pollution due to the data availability. Based on the nitrogen oxides emission per rural person in the manual and the effective use rates of fertilizer in the yearbook, the distributions of temporal nitrogen oxides emission data over the study period. The nitrogen oxides emission data were used to obtain the land cover indicator (LCI) for the subsequent calculation of the PNPI. All processing was performed in ArcGIS and Matlab.

#### 4.2.4. Summing the scores

**Figure 4:**
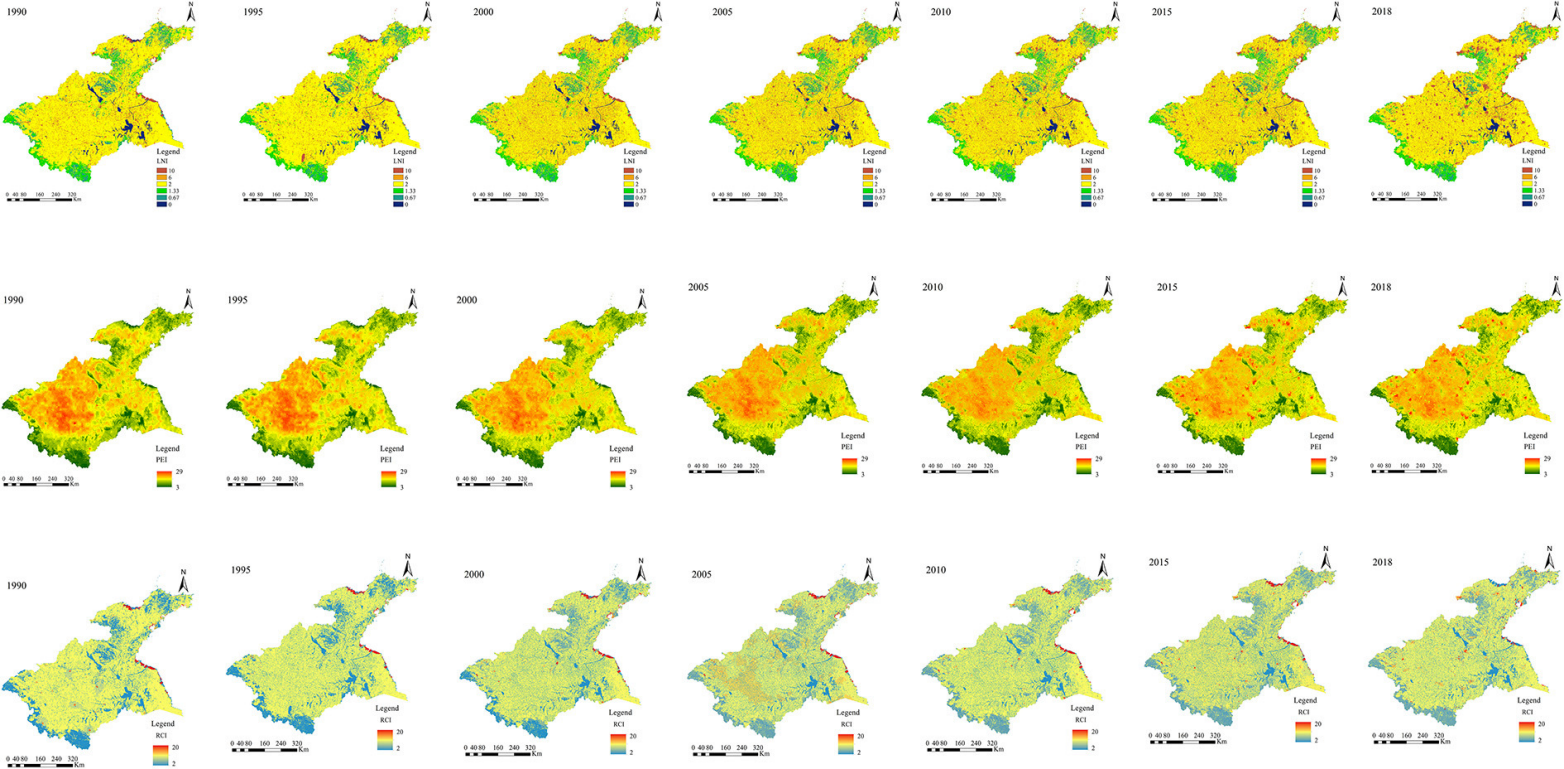
Spatiotemporal distribution of LNI, RCI, and PEI in the Huaihe River Basin over the 1990–2018 period.

**Figure 5:**
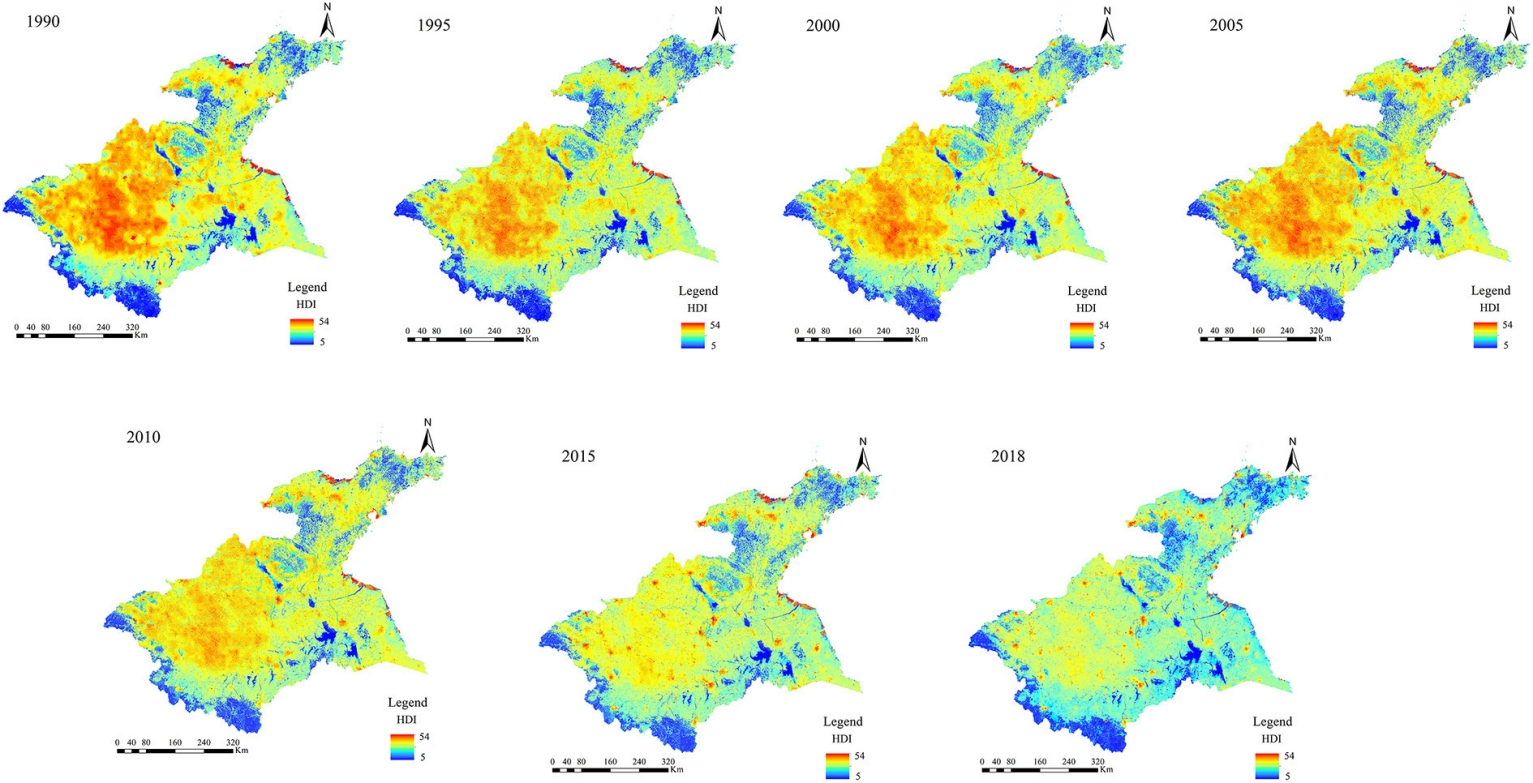
Spatiotemporal distribution of HDI in the Huaihe River Basin over the 1990–2018 period.

The spatial distributions of LNI, RCI, and PEI in the Huaihe River Basin were obtained by summing the above-attributed values in ArcGIS 10.3 software (Figure 4), whereas the HDI values were obtained using Eq. 1 (Figure 5).

### 4.3. Spatiotemporal analysis of the human disturbances

#### 4.3.1. Spatial centroid

The centroidal dynamics can reflect the spatial transformation characteristics and trends of regional elements (29). In this study, the centroid method was used to analyze the spatiotemporal distribution of the HDI values in the Huaihe River Basin according to the following equations:


(2) $$\begin{array}{l} X=\frac{\sum_{i=1}^{n}\sum_{j=1}^{m}\left(x_{ij}\times HDI_{ij}\right)}{\sum_{i=1}^{n}\sum_{j=1}^{m}HDI_{ij}};\\ Y=\frac{\sum_{i=1}^{n}\sum_{j=1}^{m}\left(y_{ij}\times HDI_{ij}\right)}{\sum_{i=1}^{n}\sum_{j=1}^{m}HDI_{ij}}\backslash n \end{array}$$

where *X* and *Y* denote the horizontal and vertical coordinates of the centroid, respectively; *n* and *m* denote the numbers of rows and columns of the raster map, respectively; *x*_*ij*_ and *y*_*ij*_ denote the latitude and longitude coordinates of row *i* and column *j*, respectively; *HDI*_*ij*_ denotes the value of HDI in row *i* and column *j* of the grid.

#### 4.3.2. Spatial pattern analysis of HDI

In order to explore the spatial distribution of HDI in the Huaihe River Basin, the global Moran's I index was used to determine whether the HDI values are spatially clustered. In addition, the Getis-Ord Gi^*^ index was used to further describe spatial distributions of human disturbances according to the following equations:


(3) $$\begin{array}{lll} I & = & \frac{\sum_{i=1}^{n}\sum_{j=1}^{n}W_{ij}\left(x_{i}-\bar{x}\right)\left(x_{j}-\bar{x}\right)}{S^{2}\sum_{i=1}^{n}\sum_{j=1}^{n}W_{ij}}\;\backslash n \end{array}$$

(4) $$\begin{array}{lll} G^{*} & = & \frac{\sum_{j=1}^{n}W_{ij}\left(d\right)x_{j}}{\sum_{j=1}^{n}x_{j}}\backslash n \end{array}$$

where *I* denote the global Moran's *I*; *n* denotes the number of data; *x*_*i*_ and *x*_*j*_ denote the HDI values of the grid *i* and *j*, respectively; *W*_*ij*_ denotes the spatial weight matrix. In addition, the *Z* test was performed on Gi^*^ to assess the reliability of the results. A positive *Z* (Gi^*^) value indicates a higher value around position *i* than the average value, suggesting a high-value spatial concentration (hot spot area). A negative *Z* (Gi^*^) value indicates a low-value spatial concentration (hot spot area).

## 5. Results

### 5.1. Spatiotemporal changes in LNI, RCI, and PEI

The three types of human disturbances showed different spatiotemporal changes in the Huaihe River Basin over the 1990–2018 period (Figure 4).

The areas with high naturalness were mainly located in the southwestern and western parts of the midstream, as well as in the Shandong Peninsula and northeastern parts of Yishusihe. These areas are mainly dominated by forestland, grassland, and water bodies. However, areas with high naturalness exhibited a certain spatiotemporal reduction trend. Regions with low naturalness values were observed in the midstream and Yishusihe, where urban, rural, and arable lands are the main land use types. Indeed, these areas tend to cluster gradually, resulting in a large combined area.

Areas with high resource consumption were mainly concentrated in the Jiangsu coastal zone and the northern part of the Shandong Peninsula, where urban land is the main land use type. In addition, relatively high resource consumption was observed from 2005 in the midstream and Yishusihe, while scattered point areas with high resource consumption were observed in the study area. Areas with low resource consumption were mainly found in forest land, grassland, and water bodies in the western and southwestern parts of the study area, showing a gradually decreasing spatiotemporal trend.

Pollutant emissions showed a downward trend over the study period. Before 2005, areas with high pollutant emissions are mainly concentrated in the arable land of the midstream, Shandong Peninsula, and Yishusihe, showing a slightly decreasing spatiotemporal trend. Areas with low pollutant emissions were mainly concentrated in the Jiangsu coastal zone, the southwestern, western, and southeastern parts of the midstream, and the northeastern part of the Shandong Peninsula, while no substantial spatiotemporal differences in the pollutant emissions were observed. It should be noted that there were increases in pollutant emissions in some parts of the study area from 2010, showing scattered areas.

### 5.2. Spatiotemporal changes in human disturbances

**Figure 6:**
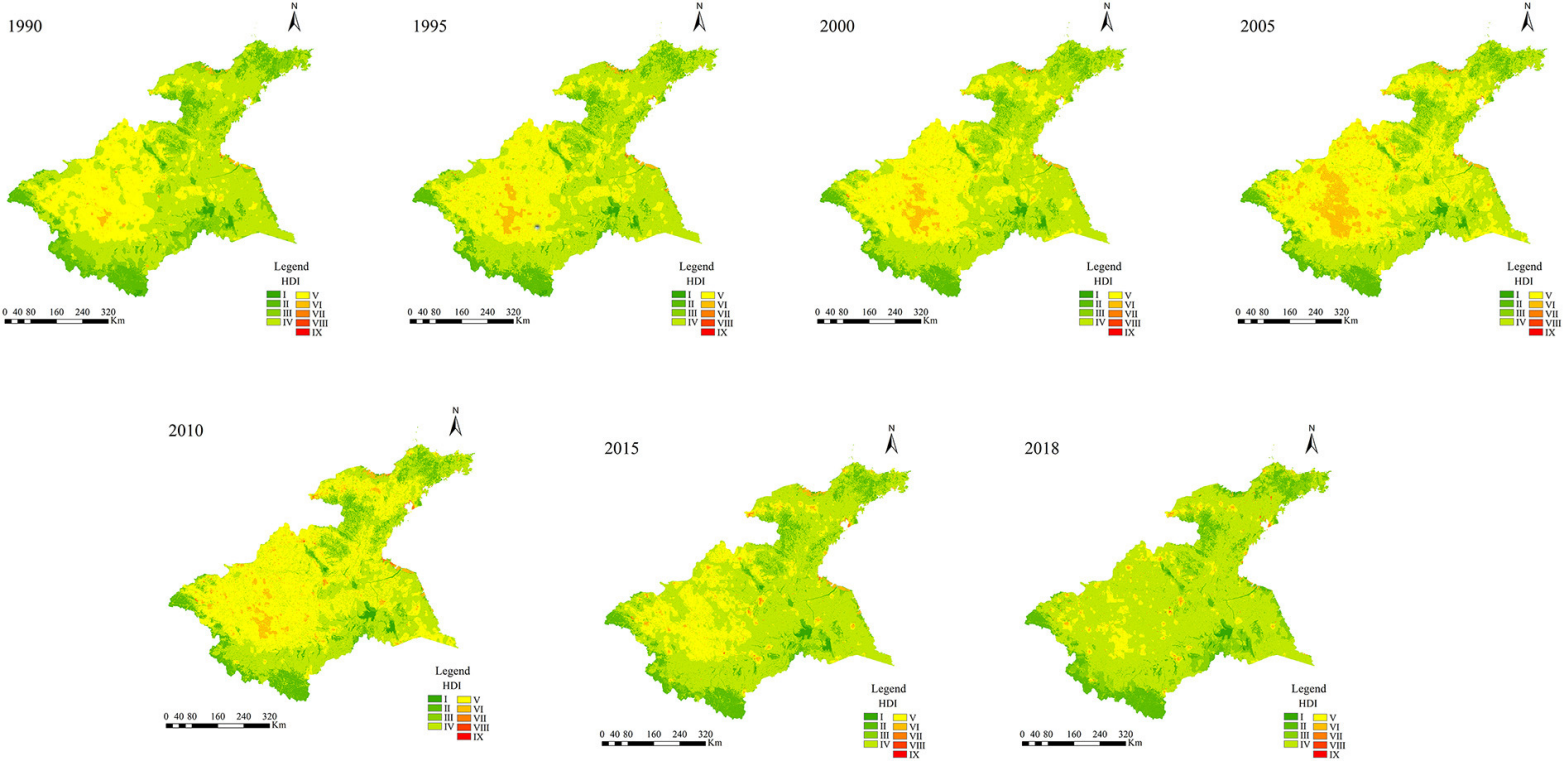
Spatiotemporal distribution of different levels of the HDI in the Huaihe River Basin over the 1990–2018 period.

The spatial distributions of human disturbances and classification results in the study area are shown in Figures 5, 6, respectively. The results showed substantial spatiotemporal differences in human disturbance levels over the 1990–2018 period. These changes in human disturbances can be divided into two distinct phases, namely 1990–2005 and 2010–2018. The 1990–2005 period corresponded to the expansion period of high-level human disturbances. The levels of HDI in the coastal zones of Jiangsu and the northern part of the Shandong Peninsula were considerably higher than those in other regions. The spatial range of areas with levels V and VI expanded substantially to the central part of the midstream and Yishusihe, showing the highest areas with level VI in 2005. On the other hand, areas with low-level human disturbances began to expand in the 2010–2018 period. Indeed, an obvious reduction in areas with levels VI was observed in the midstream, while areas with level V gradually decreased, increasing areas with the HDI IV level. In addition, other areas with level V of the HDI were almost completely replaced by those with level IV of the HDI, while only some areas with levels V-IX of the HDI were observed mainly around the city areas in the study area.

The percentages of grids for different HDI levels in the study area were obtained from 1990 to 2018 (Table 4). According to the obtained results, levels IV and V of HDI exhibited the highest percentages of grids in the Huaihe River Basin over the study period. Areas with levels II to IV decreased first and then increased over the 1990–2018 period, while areas with levels V to VIII showed the opposite change. The highest intensity of human disturbances was observed in 2005. In addition, areas with levels II to IV and V to VIII accounted for 52.52 and 48.41% of the total surface of the study area, respectively, whereas the lowest intensity of human disturbances was observed in 2018. In this year, areas with levels II, III, and IV accounted for 89.76% of the total surface of the study area, while areas with levels V to VIII accounted for only 9.72% of the total surface of the study area. The obtained results showed an increasing-decreasing temporal trend in the intensity of human disturbances in the study area.

**Table 4:** Different levels of HDI in the Huaihe River Basin over the 1990–2018 period (%).

**Study area**	**Year**	**I**	**II**	**III**	**IV**	**V**	**VI**	**VII**	**VIII**
The Huaihe River Basin	1990	0.59	11.11	13.98	45.59	27.43	1.18	0.10	0.01
	1995	0.48	11.45	8.46	45.39	31.46	2.51	0.22	0.02
	2000	0.42	9.74	8.44	43.70	34.19	3.23	0.23	0.05
	2005	0.42	9.72	7.93	33.44	41.62	6.45	0.34	0.07
	2010	0.57	10.27	7.33	32.92	45.47	2.89	0.47	0.07
	2015	0.68	10.84	9.50	57.22	19.73	1.60	0.39	0.04
	2018	0.50	10.60	14.59	64.57	8.28	0.93	0.45	0.06
The Upstream	1990	0.03	18.41	22.80	38.26	20.39	0.12	0.01	0.00
	1995	0.02	20.28	9.31	44.97	24.85	0.50	0.07	0.00
	2000	0.02	17.07	9.86	46.36	26.27	0.37	0.05	0.00
	2005	0.04	16.79	7.66	40.71	33.14	1.54	0.11	0.01
	2010	0.13	17.49	5.91	43.52	32.14	0.71	0.10	0.02
	2015	0.13	17.03	12.24	58.50	11.43	0.54	0.12	0.02
	2018	0.07	18.99	20.86	55.71	3.73	0.39	0.22	0.03
Midstream	1990	0.85	11.17	9.58	34.70	42.12	1.50	0.07	0.01
	1995	0.79	11.11	5.58	34.83	43.19	4.27	0.21	0.03
	2000	0.59	10.43	4.77	32.33	45.64	5.92	0.23	0.07
	2005	0.68	10.28	4.80	25.15	46.10	12.54	0.37	0.08
	2010	0.81	10.63	4.63	25.48	53.83	4.19	0.36	0.06
	2015	1.03	10.74	6.69	49.05	30.88	1.18	0.37	0.05
	2018	0.95	10.73	9.97	65.16	12.05	0.70	0.36	0.08
Downstream	1990	1.60	5.86	18.24	69.81	3.57	0.88	0.04	0.00
	1995	1.17	6.96	6.98	76.60	7.30	0.83	0.16	0.00
	2000	1.38	5.85	8.33	73.64	9.57	1.15	0.09	0.00
	2005	1.28	5.62	7.49	60.55	23.38	1.58	0.10	0.00
	2010	1.88	5.56	6.57	58.03	25.98	1.66	0.25	0.07
	2015	1.95	6.12	11.80	74.82	3.77	1.27	0.23	0.04
	2018	0.73	7.05	20.96	67.80	2.94	0.41	0.10	0.01
Yishusihe	1990	0.10	6.63	11.53	49.91	30.61	1.06	0.14	0.02
	1995	0.01	6.79	11.38	41.44	38.44	1.69	0.22	0.02
	2000	0.10	4.98	11.03	41.10	40.70	1.77	0.26	0.05
	2005	0.02	4.91	11.16	25.88	54.34	3.23	0.38	0.08
	2010	0.18	5.96	10.75	26.40	54.02	2.05	0.56	0.09
	2015	0.19	7.27	10.99	60.16	19.17	1.83	0.38	0.01
	2018	0.10	6.49	12.61	71.21	7.96	1.21	0.37	0.05
Shandong Peninsula	1990	0.43	15.17	19.72	55.26	7.83	1.39	0.18	0.01
	1995	0.33	15.60	11.29	57.66	13.03	1.73	0.33	0.04
	2000	0.17	12.40	12.27	54.72	18.16	1.90	0.34	0.05
	2005	0.15	12.91	10.69	43.40	29.67	2.64	0.47	0.07
	2010	0.14	13.52	9.75	39.08	33.56	2.95	0.88	0.11
	2015	0.18	14.62	11.08	61.31	9.16	2.91	0.68	0.04
	2018	0.18	12.58	20.70	57.92	5.86	1.63	1.07	0.05

Areas with level V accounted for high proportions in the midstream and Yishusihe over the entire study period, while level IV was observed mainly in the upstream, downstream, and Shandong Peninsula. Overall, decreasing-increasing trends in levels II, III, and IV were observed in the five secondary basins of the study area, exhibiting the lowest areas in 2005 and 2010 and the highest in 2018. Levels II, III, and IV were observed mainly in the upstream, downstream, and Shandong peninsula, accounting for more than 60% of the total surface of the study area, as well as in the Yishusihe and Midstream, covering 40% of the total surface of the study area, while the areas above levels V were the opposite. These results indicated that the intensities of human disturbances in the upstream, downstream, and Shandong Peninsula were lower than those observed in the midstream and Yishusihe.

The spatial centroid variations of human disturbances in the Huaihe River Basin from 1990 to 2018 were determined using ArcGIS 10.3 based on the spatial centroid migration model (Figure 6). During the study period, the centroids of human disturbances were distributed in the junction of the midstream and Yishusihe, showing spatiotemporal migration direction. The centroid migration showed a 45° shift trend from southwest to northeast, with fluctuations in the speed of the centroid migration. The lowest speed of centroid migration was observed from 2010 to 2015. The space centroid shifted 602.80 m to the southwest, with an offset speed of 120.56 m/year. Whereas the highest speed of centroid was observed from 2015 to 2018. In this period, the space centroid shifted 3692.56 m to the northeast, with an offset speed of 738.51 m/year.

### 5.3. Cold and hot spots changes of human disturbances

**Figure 7:**
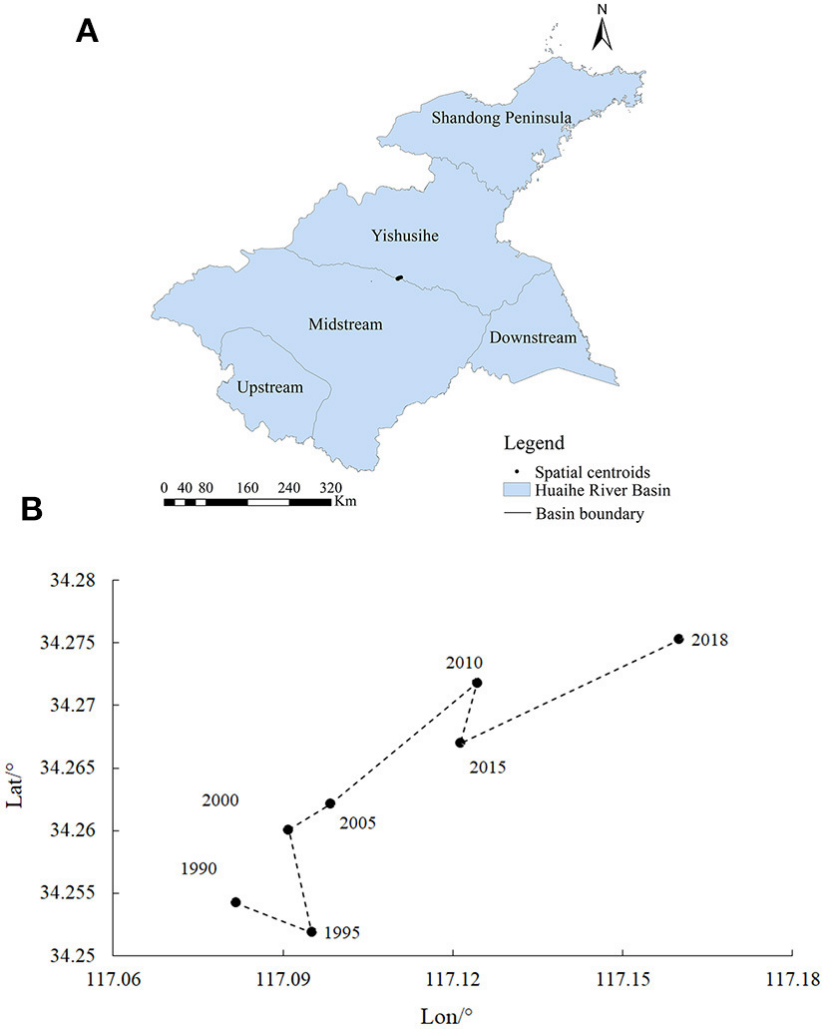
**(A, B)** Spatial distributions of cold and hot spots of the human disturbances in the study area.

To quantitatively analyze the spatiotemporal changes in human disturbances in the Huaihe River Basin over the 1990–2018 period, the global Moran's I index was calculated in this study using the spatial autocorrelation model in ArcGIS 10.3 (Table 5). The results showed significant and positive spatial autocorrelation (*p* < 0.01) in human disturbances in the Huaihe River Basin. Therefore, the Getis-Ord Gi^*^ index can be used to further investigate the evolution characteristics of the local spatial concentration of human disturbances in the study area (Figure 7). Overall, the cold and hot spots of human disturbances in the study area exhibited similar patterns during the study period. Most of the hot spots were observed in the midstream, Yishusihe Peninsula, and central part of the Shandong Peninsula, where urban, rural, and arable lands are dominant. In addition, areas with hot spots of human disturbances showed a decreasing temporal trend. Most cold spot areas were found in the upstream, western, southwestern, and southeastern of the midstream, as well as in the western and northeastern parts of the Shandong Peninsula. These areas are dominated by forest land, grassland, and water bodies. The distribution of cold spot areas was relatively stable without significant changes.

**Table 5:** Spatial autocorrelation indices of the HDI in the Huaihe River Basin.

	**1990**	**1995**	**2000**	**2005**	**2010**	**2015**	**2018**
Moran's I	0.25	0.23	0.27	0.33	0.25	0.26	0.21
*Z*	46.92	53.89	62.37	75.86	71.67	67.31	53.87
*P*-level	0.00	0.00	0.00	0.00	0.00	0.00	0.00

## 6. Discussion

### 6.1. Benefits of the HDI

Human disturbances have caused serious environmental problems, threatening sustainable human development (4, 16). Human disturbances affect ecosystems mainly through direct anthropogenic activities, such as the conversion of natural land into artificial land for economic development and urbanization, resulting in substantial consumption of resources and, consequently, serious pollution and climate change (2, 3, 58). The investigation of these human disturbances is an essential step for environmental protection and restoration. The HDI method can be used to classify the possible disturbances into three main types, namely land use naturalness transformation, resource consumption, and pollutant emissions. These three human disturbance types can be effectively quantified based on the literature (2, 3, 44, 47). Indeed, the required RS, GIS-related, multisource data in the HDI method are easily accessible, making it possible to investigate temporal changes in the HDI in several study areas with the data. Moreover, the HDI method can be applied at different scales by selecting appropriate data according to the specific characteristics of the study area, demonstrating stronger universal applicability than that of other traditional methods (e.g., biological indicators and pollution discharges). The results of the HDI calculation can support the analysis of the spatiotemporal changes in the regional human disturbances by considering the ecological and physical-geographic processes, thereby providing further insights into the relationships between humans and land, as well as the interactions between human activities and ecosystems. Therefore, the HDI method is of great importance for policy development and implementation of protective measures in the region. For areas with serious human disturbances, it is crucial to identify first the dominant human disturbances source types, then formulate corresponding policies to mitigate and control them, thereby reducing the cost, time, and difficulty of environmental protection and restoration.

### 6.2. Dynamics of human disturbances

**Figure 8:**
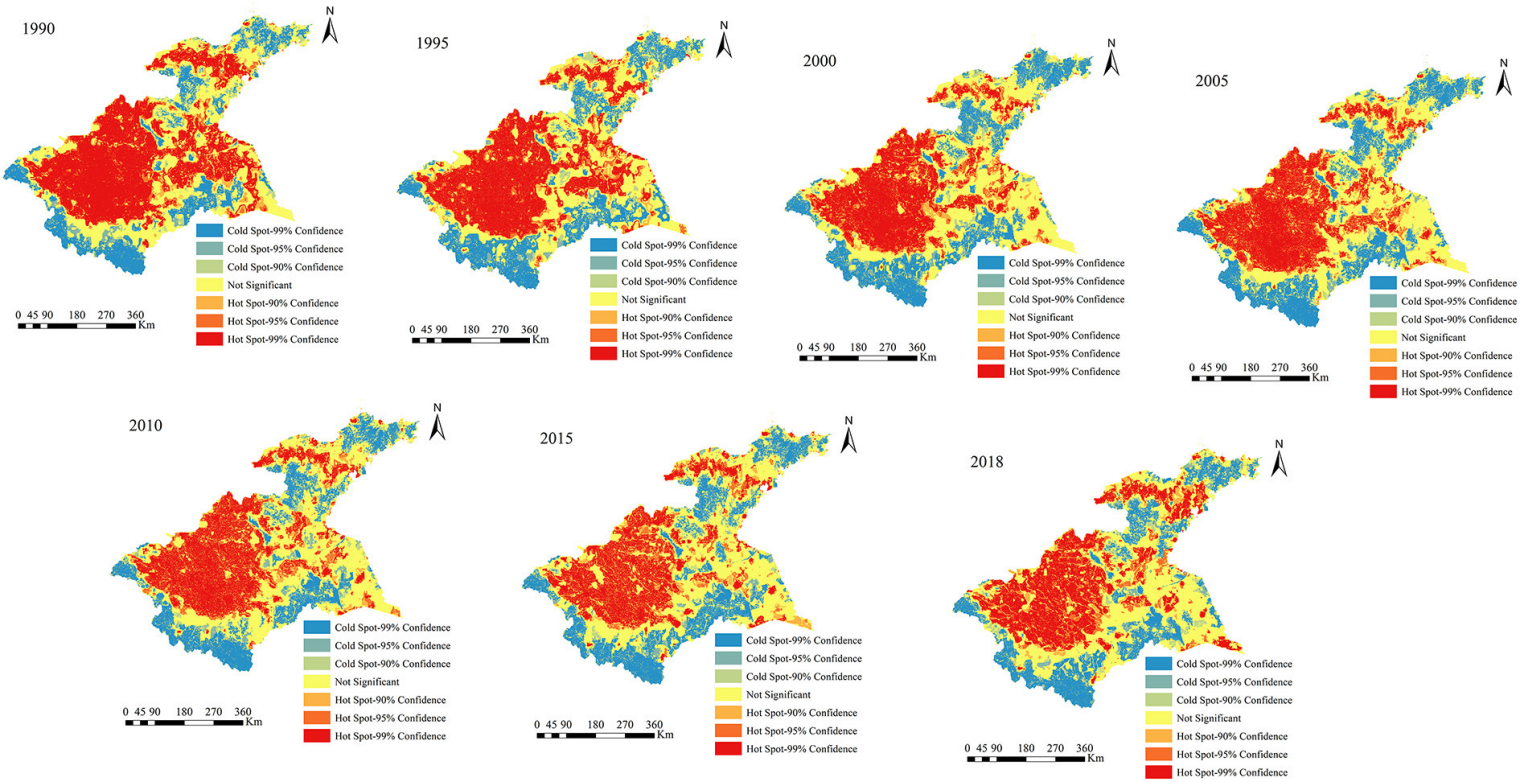
Migration of centroids of human disturbances over the 1990–2018 period.

Agriculture and industry were consistently the main types of human activities in the Huaihe River Basin over the 1995–2018 period because of the differences in levels of economic development and regional development policies. The results of this study showed spatiotemporal variations in the intensities of human disturbances in the secondary basins of the Huaihe River Basin as a result of the different levels of regional economic and policy development. The HDI results suggested two distinct periods during which considerable changes in the intensities of human disturbances in the Huaihe River Basin were observed, namely 1990–2005 and 2010–2020. The overall human disturbances in the study area showed an increasing trend over the 1990–2005 period due to the increasing economic development that marked this period. Indeed, a large area of arable land was converted to urban land, resulting in a substantial reduction of the land use naturalness in many areas (Figure 4). Moreover, considerable pollutant emissions and resource consumption were observed during the 1990–2005 period. In terms of spatial distributions, areas with high levels of HDI were mainly found in the arable and industrial lands (Figures 2, 6), providing evidence of the greater impacts of agricultural and industrial activities on the ecosystem than those of other human activities (33). On the other hand, positive effects of the environmental policies (e.g., Returning Farmland to Forest Program, Energy Saving and Emission Reduction, and Interim Regulations on the Prevention and Control of Water Pollution in the Huaihe River Basin) on reducing human disturbances were observed in the 2010–2018 period (15, 59). In addition, agricultural and industrial activities have changed from extensive to refined management, improving pesticide, fertilizer, and energy use efficiencies, while the consumption of resources and energy per unit area has been significantly reduced (28, 60). However, the increase in the urbanization level and urban population resulted in structural changes in human activities (61). In general, the levels of human disturbances in agricultural and industrial areas decreased to a certain extent, while those in urban areas of the inland plain increased substantially (Figure 6). The intensity of human disturbances decreased in the Huaihe river Basin (Figure 8), which might be due to the overall influences of improved management efficiency and pollution control capacity in the study area (12, 17). The intensities and spatial distributions of human disturbances are closely related to the urbanization process, as well as to the intensity of resource exploitation and utilization in the Huaihe river Basin. Therefore, government strategies and programs play a crucial role in the spatiotemporal changes of human disturbances (10, 62, 63).

### 6.3. Future research

The results of the present study demonstrated the effectiveness of the HDI method in investigating human disturbances in the Huaihe River Basin. However, due to our limited understanding of the complexity of human disturbances, all the types of human activities were not considered in the HDI method, while the combined effects of human disturbances were not demonstrated in the present study. Besides analyzing human disturbances gradients, policymakers and scholars can also combine the natural environmental characteristics, natural resource endowment, and the regional political and economic context to determine the thresholds of human disturbances that the study area can tolerate (58, 64). These suggestions may contribute to formulating and optimizing policies to effectively reduce the intensities of human disturbances and maintain the sustainable development of human society. In the future, dynamic modules, such as the conversion process of land use, consumption of natural resources and energy, and migration and transformation processes of pollutants, need to be considered in the HDI method, with the support of big data, to more accurately quantify and monitor human disturbances, thereby providing some degree of prediction capability for the future change of human disturbances.

## 7. Conclusions

In this study, three components of human disturbances were quantified, namely land use naturalness transformation, natural resource consumption, and pollutant emissions, using the land use naturalness index (LNI), resource consumption index (RCI), and pollution emission index (PEI). The intensities of human disturbances were calculated using the HDI method by combining RS, GIS, and multisource data. The HDI method is based on the spatiotemporal variabilities of input parameters, achieving more convenient and comprehensive dynamic monitoring and evaluation of human disturbances. Moreover, this method is applicable to research areas of different scales. The results of this demonstrated the effectiveness and applicability of the HDI method to quantify human disturbances in the Huaihe River Basin, showing good results. The results of this study showed gradual increases and decreases in human disturbances in the Huaihe River Basin in the 1990–2005 and 2010–2018 periods, respectively. The intensity and spatial distribution of human disturbances were closely related to the urbanization process and intensities of resource exploitation and utilization, which are mainly determined through government strategies and economic development. In the early period (1990–2005), agricultural and industrial activities were the main causes of the higher intensity of regional human disturbances in the midstream and Yishusihe and Shandong Peninsula than that in other areas. Whereas the results showed decreases in the intensity of human disturbances in arable and industrial lands in the 2010–2018 period as a result of the implementation of several regional programs, such as Returning Farmland to Forest and Energy Saving and Emission Reduction programs. However, rapid population growth and accelerated urbanization resulted in changes in the structure of human activities, substantially increasing the intensity of human disturbances in urban areas. In general, the intensity of regional human disturbances can be effectively reduced by improving the energy structure and utilization, as well as minimizing pollutant emissions. In addition, maintaining grassland, forest land, and water body areas plays an important role in sustaining ecosystem naturalness and reducing the intensity of regional human disturbances. The results of the present study provide a scientific basis for ensuring effective environmental protection of the Huaihe River Basin, as well as a novel method for quantifying human disturbances.

## Data availability statement

The original contributions presented in the study are included in the article/supplementary material, further inquiries can be directed to the corresponding authors.

## Author contributions

HW and YZ designed the research. HW drafted the manuscript. MZ, CW, and KW analyzed the data and prepared figures. YZ and WS discussed the results and revised the manuscript. All authors contributed to the article and approved the submitted version.

## References

[1] Woodward RT Wui YS. The economic value of wetland services: a meta analysis. Ecol Econ. (2001) 37:257–70. 10.1016/S0921-8009(00)00276-7

[2] Sanderson EW Jaiteh MS Levy MA Redford K Wannebo AV Woolmer G. The human footprint and the last of the wild. Bioscience. (2002) 52:891–904. 10.1641/0006-3568(2002)052[0891:THFATL]2.0.CO;2

[3] Brown MT Vivas MB. Landscape development intensity index. Environ Monit Assess. (2005) 101:289–309. 10.1007/s10661-005-0296-6 15736887

[4] Zalasiewicz J Williams M Steffen W Crutzen P. The new world of the Anthropocene. Environ Sci Technol. (2010) 44:2228–31. 10.1021/es903118j 20184359

[5] Sun Y Zhao D Wu T Wei B Gao S Li Y . Temporal and spatial dynamic changes and landscape pattern response of Hemeroby in Dayang estuary of Liaoning Province, China. Artic Acta Ecol Sin. (2012) 32:3645–55. 10.5846/stxb201112061865

[6] Tian Y Liu B Hu Y Xu Q Qu M Xu D. Spatio-temporal land-use changes, and the response in landscape pattern to hemeroby in a resource-based city. ISPRS Int J Geo-Inf. (2020) 9:20. 10.3390/ijgi9010020

[7] Bhat SU Khanday SA Islam ST Sabha I. Understanding the spatiotemporal pollution dynamics of highly fragile montane watersheds of Kashmir Himalaya, India. Environ Pollut. (2021) 286:117335. 10.1016/j.envpol.2021.117335 34051690

[8] Vorosmarty CJ McIntyre PB Gessner MO Dudgeon D Prusevich A Green P . Global threats to human water security and river biodiversity. Nature. (2010) 467:555–61. 10.1038/nature09440 20882010

[9] Balter M. Archaeologists say the “Anthropocene” is here-But it began long ago. Science. (2013) 340:261–2. 10.1126/science.340.6130.261 23599452

[10] Zhou Y Ning L Bai X. Spatial and temporal changes of human disturbances and their effects on landscape patterns in the Jiangsu coastal zone, China. Ecol Indic. (2018) 93:111–22. 10.1016/j.ecolind.2018.04.076

[11] Ellis EC Gauthier N Goldewijk KK Bird RB Boivin N Diaz S . People have shaped most of terrestrial nature for at least 12,000 years. Proc Natl Acad Sci. (2021) 118:e2023483118. 10.1073/pnas.2023483118 33875599 PMC8092386

[12] Wang J Zhou Y Bai X Li W. Effect of algal blooms outbreak and decline on phosphorus migration in Lake Taihu, China. Environ Pollut. (2022) 296:118761. 10.1016/j.envpol.2021.118761 34971742

[13] Johnson BG Zuleta GA. Land–use land–cover change and ecosystem loss in the Espinal ecoregion, Argentina. Agric Ecosyst Environ. (2013) 181:31–40. 10.1016/j.agee.2013.09.002

[14] Turner MG Gardner RH O'Neill RV. Landscape disturbance dynamics. In:Turner MG Gardner RH, editors. Landscape Ecology in Theory and Practice. New York, NY: Springer (2015). p. 10–25. 10.1007/978-1-4939-2794-4_6

[15] Wang H Zhang M Wang C Wang K Wang C Li Y . Spatial and temporal changes of landscape patterns and their effects on ecosystem services in the Huaihe River Basin, China. Land. (2022) 11:513. 10.3390/land11040513

[16] Costanza R D'Arge R de Groot R Farber S Grasso M Hannon B . The value of the world's ecosystem services and natural capital. Nature. (1997) 387:253–60. 10.1038/387253a0

[17] Liu H Gao C Wang G. Understand the resilience and regime shift of the wetland ecosystem after human disturbances. Sci Total Environ. (2018) 643:1031–40. 10.1016/j.scitotenv.2018.06.276 30189520

[18] Bush MB Miller MC Oliveira PD Colinvaux PA. Two histories of environmental change and human disturbance in eastern lowland Amazonia. Holocene. (2000) 10:543–54. 10.1191/095968300672647521

[19] Brentrup F Küsters J Lammel J Kuhlmann H. Life cycle impact assessment of land use based on the hemeroby concept. Int J Life Cycle Assess. (2002) 7:339–48. 10.1007/BF02978681

[20] Tang T Stevenson RJ Grace JB. The importance of natural versus human factors for ecological conditions of streams and rivers. Sci Total Environ. (2019) 704:135268. 10.1016/j.scitotenv.2019.135268 31810677

[21] Qin H Cui L Cao XLQ Chen T. Evaluation of the Human interference on the microbial diversity of poyang lake using high-throughput sequencing analyses. Int J Environ Res Public Health. (2019) 16:4218. 10.3390/ijerph16214218 31671714 PMC6861916

[22] Zou L Liu Y Wang Y Hu X. Assessment and analysis of agricultural non-point source pollution loads in China: 1978–2017. J Environ Manage. (2020) 263:110400. 10.1016/j.jenvman.2020.110400 32174536

[23] Caplat P Lepart J Marty P. Landscape patterns and agriculture: modelling the long-term effects of human practices on Pinus sylvestris spatial dynamics (Causse Mejean, France). Landscape Ecol. (2006) 21:657–70. 10.1007/s10980-005-4430-1

[24] Enzenhofer K Mayrhofer S. Hemerobie der Wälderim Biosphärenpark Wienerwald. Verh Zool Bot Ges Österreich Wein. (2009) 146:1–16. 10.25365/thesis.6003

[25] Jalas J. Hemerobe und hemerochore Pflanzenarten. Ein terminologischer Reformversuch. Acta Soc Fauna Flora Fenn. (1955) 72:1–15.

[26] Xiao C Jie X Wu T Jiang G Bian H Xu W. Dynamic changes of landscape pattern and hemeroby in Xiamen island wetland, Zhejiang province, China. Chin J Appl Ecol. (2014) 25:3255–62. 10.13287/j.1001-9332.2014.0188 25898624

[27] Stankowich T. Ungulate flight responses to human disturbance: a review and meta-analysis. Biol Conserv. (2008) 141:2159–73. 10.1016/j.biocon.2008.06.026

[28] Fan X Dai X Yang G Jia Z Liu L Sun N. Detecting artificialization process and corresponding state changes of estuarine ecosystems based on naturalness assessment. Ocean Coast Manage. (2017) 146:178–86. 10.1016/j.ocecoaman.2017.07.007

[29] Yuan S Zhu C Yang L Xie F. Responses of ecosystem services to urbanization-induced land use changes in ecologically sensitive suburban areas in Hangzhou, China. Int J Env Res Pub He. (2019) 16:1124. 10.3390/ijerph16071124 30925834 PMC6480383

[30] Wu T Zha P Yu M Jiang G Zhang J You Q . Landscape pattern evolution and its response to human disturbance in a newly metropolitan area: a case study in Jin-Yi Metropolitan Area. Land. (2021) 10:767. 10.3390/land10080767

[31] Günlü A Kadiogullari AI Keleş S Başkent EZ. Spatiotemporal changes of landscape pattern in response to deforestation in Northeastern Turkey: a case study in Rize. Environ Monit Assess. (2009) 148:127–37. 10.1007/s10661-007-0144-y 18240002

[32] Chen A Zhu B Chen L Wu Y Sun R. Dynamic changes of landscape pattern and eco-disturbance degree in Shuangtai estuary wetland of Liaoning Province, China. Chin J Appl Ecol. (2010) 21:1120–8. 10.13287/j.1001-9332.2010.0191 20707090

[33] Li H Man W Li X Ren C Wang Z Li L . Remote sensing investigation of anthropogenic land cover expansion in the low elevation coastal zone of Liaoning Province, China. Ocean Coast Manage. (2017) 148:245–59. 10.1016/j.ocecoaman.2017.08.007

[34] Yorkina NV Podorozhniy SM Velcheva LG Honcharenko YV Zhukov OV. Applying plant disturbance indicators to reveal the hemeroby of soil macrofauna species. Biosyst Divers. (2020) 28:181–94. 10.15421/012024

[35] Wang K Chu D Yang Z. Flood control and management for the transitional Huaihe River in China. Procedia Eng. (2016) 154:703–9. 10.1016/j.proeng.2016.07.572

[36] Pan Z Ruan X Qian M Hua J Shan N Xu J. Spatio-temporal variability of streamflow in the Huaihe River basin, China: climate variability or human activities. Hydrol Res. (2018) 49:177–93. 10.2166/nh.2017.155

[37] Kowalski MF Krausmann F Pallua I. A socio-metabolic reading of the Anthropocene: modes of subsistence, population size, and human impact on Earth. Anthropocene Rev. (2014) 1:8–33. 10.1177/2053019613518033

[38] Cincotta RP Engelman R. Nature's Place: Human Population Density and the Future of Biological Diversity. Washington, DC: Population Action International, America. (2000) 6:275. 10.1071/PC000275

[39] Elvidge CD Baugh KE Kihn EA Kroehl HW Davis ER Davis DW. Relation between satellite-observed visible-near infrared emissions, population, economic activity and electric power consumption. Int J Remote Sens. (1997) 18:1373–9. 10.1080/014311697218485

[40] Reiss KC Hernandez E Brown MT. Application of the landscape development intensity (LDI) index in wetland mitigation banking. Ecol Model. (2014) 271:83–9. 10.1016/j.ecolmodel.2013.04.017

[41] Walz U Stein C. Indicators of hemeroby for the monitoring of landscapes in Germany. J Nat Conserv. (2014) 22:279–89. 10.1016/j.jnc.2014.01.007

[42] Fan J Li PX. The scientific foundation of major function oriented zoning in China. J Geogr Sci. (2009) 19:515–31. 10.1007/s11442-009-0515-031021443

[43] Tang H Wu W Yang P Chen Y Verburg PH. Recent progresses of land use and land cover change (LUCC) models. Acta Geogr Sin. (2009) 64:456–68.

[44] Xu Y Xu X Tang Q. Human activity intensity of land surface: concept, methods and application in China. J Geogr Sci. (2016) 26:1349–61. 10.1007/s11442-016-1331-y

[45] Levin N Kark S Crandall D. Where have all the people gone? Enhancing global conservation using night lights and social media. Ecol Appl. (2015) 25:2153–67. 10.1890/15-0113.1 26910946

[46] Li H Peng J Liu Y Hu Y. Urbanization impact on landscape patterns in Beijing City, China: a spatial heterogeneity. Ecol Indic. (2017) 82:50–60. 10.1016/j.ecolind.2017.06.032

[47] Munafo M Cecchi G Baiocco F Mancini L. River pollution from non-point sources: a new simplified method of assessment. J Environ Manage. (2005) 77:93–8. 10.1016/j.jenvman.2005.02.016 15990217

[48] Ye T Gabriele C Markus D. A multi-criteria model selection protocol for practical applications to nutrient transport at the catchment scale. Water. (2015) 7:2851–80. 10.3390/w7062851

[49] Liu Q. Review on environmental risk from non-point source pollution by nitrogen and phosphorus in Farmland. Chin J Soil Sci. (2016) 47:1506–13. 10.19336/j.cnki.trtb.2016.06.33

[50] Ji H Yoon C Donigian JAS Jung K. Development of the HSPF-Paddy model to estimate watershed pollutant loads in paddy farming regions. Agr Water Manage. (2007) 90:75–86. 10.1016/j.agwat.2007.02.006

[51] Nasr A Bruen M Jordan P Moles R Kiely G Byrne P. A comparison of SWAT, HSPF and SHETRAN/GOPC for modelling phosphorus export from three catchments in Ireland. Water Res. (2007) 41:1065–73. 10.1016/j.watres.2006.11.026 17258266

[52] Yang S Dong G Zheng D Xiao H Gao Y Lang Y. Coupling Xinanjiang model and SWAT to simulate agricultural non-point source pollution in Songtao watershed of Hainan, China. Ecol Model. (2011) 222:3701–17. 10.1016/j.ecolmodel.2011.09.004

[53] Pan Y Li N Zheng J Yin S Li C Yang J . Emission inventory and characteristics of anthropogenic air pollutant sources in Guangdong Province. Acta Sci Circumstantiae. (2015) 35:2655–69. 10.13671/j.hjkxxb.2014.1058

[54] Zhou Y Ma Z Wang L. Chaotic dynamics of the flood series in the Huaihe River Basin for the last 500 years. J Hydrol. (2002) 258:100–10. 10.1016/S0022-1694(01)00561-3

[55] Zhang Y You W. Social vulnerability to floods: a case study of Huaihe River Basin. Nat Hazards. (2014) 71:2113–25. 10.1007/s11069-013-0996-0

[56] Chen J Ouyang Z Zheng H Xu W. Ecosystem characteristics and regionalization of vulnerable ecological region of Huaihe River Basin. China Popul Resour Environ. (2010) 20:169–74. 10.3969/j.issn.1002-2104.2010.10.029

[57] Yang M Chen X Cheng C. Hydrological impacts of precipitation extremes in the Huaihe River Basin, China. Springerplus. (2016) 5:1731. 10.1186/s40064-016-3429-1 27777866 PMC5053955

[58] Ge J Liu Q Zan BL Lin Z Lu S Qiu B . Deforestation intensifies daily temperature variability in the northern extratropics. Nat Commun. (2022) 13:5955. 10.1038/s41467-022-33622-0 36216833 PMC9550804

[59] Zhu S Zhu L Zhao X Wang W Zhang W. A review of China's climate policies and actions since the launch of the 12th Five Year Plan. Chin J Popul Resour. (2020) 30:1–8. 10.12062/cpre.20200332

[60] Zang S Wu C Liu H Na X. Impact of urbanization on natural ecosystem service values: a comparative study. Environ Monit Assess. (2011) 179:575–88. 10.1007/s10661-010-1764-1 22013594

[61] Li D Zou Q Zhang Z. A new assessment method of sustainable water resources utilization considering fairness-efficiency-security: a case study of 31 provinces and cities in China. Sustain Cities Soc. (2002) 81:103830. 10.1016/j.scs.2022.103839

[62] Wellmann T Haase D Knapp S Salbach C Selsam P Lausch A. Urban land use intensity assessment: the potential of spatio-temporal spectral traits with remote sensing. Ecol Indic. (2018) 85:190–203. 10.1016/j.ecolind.2017.10.029

[63] Fernandez C Spayd J Brooks RP. Landscape indicators and ecological condition for mapped wetlands in Pennsylvania, USA. Wetlands. (2019) 39:705–16. 10.1007/s13157-018-1116-4

[64] Rojstaczer S Sterling SM Moore NJ. Human appropriation of photosynthesis products. Science. (2001) 294:2549–52. 10.1126/science.1064375 11752576

